# Supplementary material for: The Mastery Rubric for Bioinformatics: A tool to support design and evaluation of career-spanning education and training
Source: PLoS One. 2019 Nov 26;14(11):e0225256. doi: 10.1371/journal.pone.0225256 (PMC6879125; doi:10.1371/journal.pone.0225256)
Supplement: S1 File — (DOCX) [file pone.0225256.s001.docx]

**S1 File. Supplemental Materials**: Cognitive Task Analysis Methodology (Table A) and Figure (Figure A) with explanatory text (Text A), and Competencies (Table B).

Table A outlines the five general steps in cognitive task analysis (see Clark et al., 2008 [1]; p.580). The table describes the relevant tasks, and theoretical and literature contributions. The process by which cognitive task analysis Steps 1-4 were followed is illustrated in Figure S1.

Table A. Cognitive task analysis

| cognitive task analysis **steps^1^:** | **Tasks** | **Theoretical contributions** | **From the literature** |
| --- | --- | --- | --- |
| 1. Collect preliminary knowledge/information | Evaluation of competencies; evaluation of Blooms-level requirements | Bloom et al.,  Ambrose et al.,  Messick,  Knowles et al. | Models of the scientific method, consensus-based competencies |
| 2. Identify knowledge representations and organizations | Guild structure; career stage (stages of degrees) | Bloom’s, Messick | Scientific reasoning models (Bishop & Talbot; Wild & Pfannkuch) |
| 3. Elicit knowledge | Standard setting with bioinformaticians | Bloom’s, Messick | Curriculum & training/workforce development considerations |
| 4. Analyze and verify data | Initial range-finding, pinpointing, alignment of KSAs, stages, and other PLDs; validation with andragogy and alignment of KSAs and competencies (validation). Iterative analysis and revision of PLDs by all co-authors. | | |
| 5. Format results | Following format for Mastery Rubric construct, iterative refinement of PLDs to ensure clarity and relevance. | | |

Notes: Adapted from Clark et al. 2008 [1]; p.580. Other references in this table are included in main document.


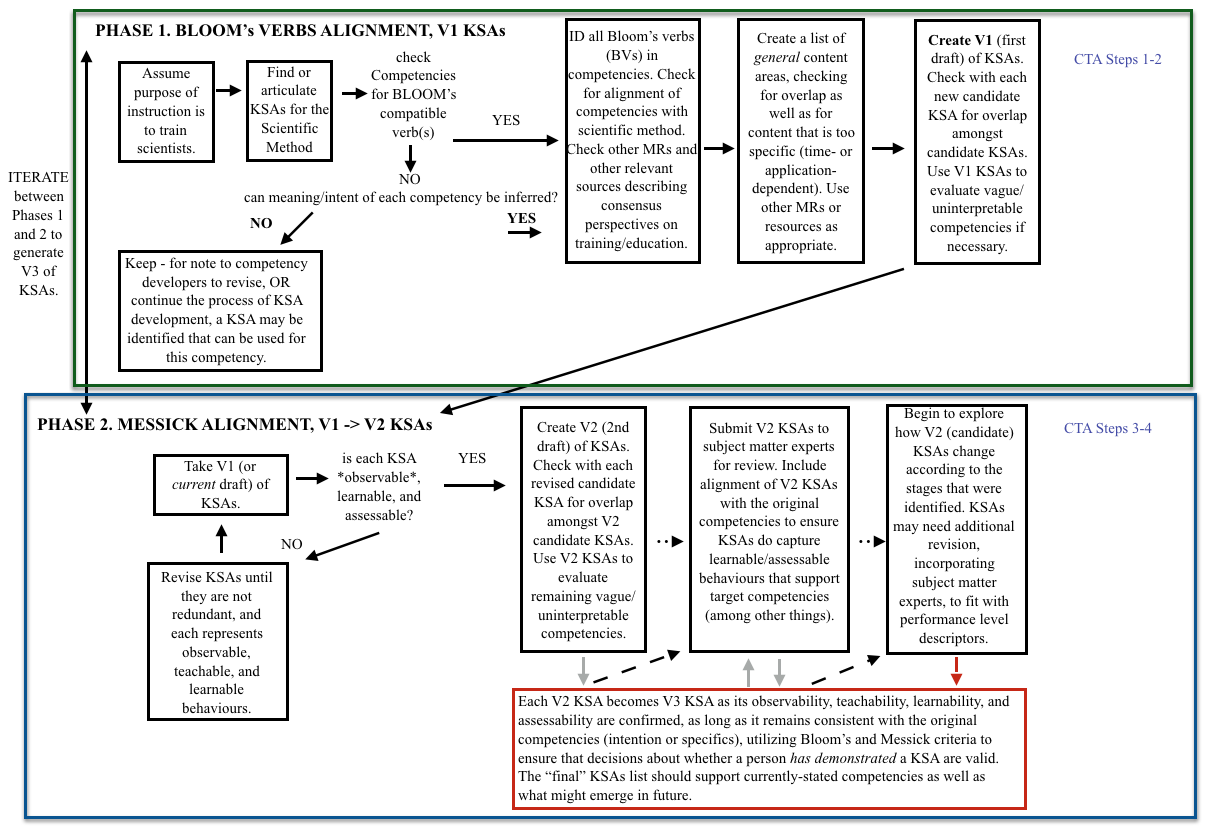


**Figure A** Two-phase approach to cognitive task analysis steps 1-4 for KSA extraction.

**Text A**. Discussion of two-phase KSA extraction with examples.

The general method (shown in Figure A) of applying cognitive task analysis (Table A) to extract KSAs from/articulate KSAs for competencies (shown in Table B) is broken into two phases (cognitive task analysis step 5 (formatting results)) follows the Mastery Rubric structure:

**Phase 1**: Assume that instruction should be underpinned by the scientific method. Beginning with the eight KSAs that support this, assess consensus-based competencies in order to align general scientific steps with disciplinary specifics. Extract actionable verbs from the competencies to formulate a list of cognitive behaviors that can be applied to relevant content in order to achieve the focus of each competency. There may be a different number of KSAs and competencies.

**i)** Read the competency;

**ii)** Identify the Bloom’s verbs;

**a)** If there are *no* Bloom’s verbs, try to infer some. If Bloom’s verbs cannot be inferred, set the competency aside to notify the competency author(s), and in case additional information comes out of further cognitive task analysis that helps “rescue” the competency.

**b)** If there *are* Bloom’s verbs, identify all that apply to that competency. Iteratively utilize the original competencies framework, other Mastery Rubrics, other consensus-based or theoretical frameworks for similar competencies, as relevant, to ensure a good match of all relevant verbs to the competency in question. Ensure that the list of verbs is not redundant.

**iii)** Create (or add the topic of the competency to) a list of general content to which the Bloom’s verbs apply. Ensure that representation of the content is concrete (because development of the cognitive ability to perform to a target level with that content will become part of the PLDs that are drafted once KSAs have been identified). NB: **Content** to which the cognitive behaviors (Bloom’s verbs) are applied is considered here as it supports a non-redundant list of KSAs that captures what the competency framers intended to support achieving for learners. However, KSAs need to be fairly generic (i.e., should not reference time- or sub-area dependent *specific* content such as specific software, methods or technology that might change rapidly over time).

**iv)** Results of **ii) b** and **iii)** are entered into the first draft (“V1”) of KSAs; as subsequent verbs and content are identified, revert to competencies that could not be immediately matched to a verb; review V1 KSAs for redundancy.

**Phase 2**: Based on this version of KSAs, revise each so that it is observable, learnable, and assessable. Refine the KSAs so that they can be described with a systematic set of PLDs following Messick’s criteria.

**v)** Considering the Messick criteria, ensure that each KSA is distinct from others, captures relevant content without becoming too specific, and is consistent with the overall intentions of the competency framers and plausible performance achievement.

**a)** Iterate between **iv)** (Phase 1) and **v)** (Phase 2) until a set of KSAs has been articulated that can be examined by subject-matter experts.

**vi)** Subject-matter experts confirm that KSAs describe what is required for the domain, and represent observable, learnable, and assessable behaviors that are relevant.

**vii)** At this point, drafting of PLDs is required to confirm that the KSAs are functional and represent the domain fully (i.e., to confirm that the cognitive task analysis is complete). PLDs are initially drafted for each KSA; if a KSA cannot be successfully described as changing in observable ways over the specified stages, then the KSA must be revised. Either PLDs must be modified so that the current version KSAs are retained, or the KSAs must be modified so that PLDs maintain distinct developmental paths *within* KSAs while ensuring consistent levels of performance for one stage *across* KSAs.

Finally, subject-matter experts review the current version of the KSAs, consulting the competencies list and external (triangulating/validating) documentation.

A specific example of identifying KSAs that are supportive of the competencies outlined in Kulikowski et al. (2012) [2] and Welch et al. (2014) [3] is given below:

***Phase 1****. Assess consensus-based competencies in order to align general scientific steps with disciplinary specifics.*

The steps of the scientific method yield a set of useful KSAs, but do not include mention of ethical practice; however competencies in both sources suggest ethical practice is a priority.

*i) Read the competency/competencies.*

There are ethics-related competencies in both documents:

Welch et al. (2014) [3]: “*Understanding* of professional, ethical, legal, security & social issues & responsibilities.”

Kulikowski et al. (2012) [2]: “*Understand and apply* knowledge in the following areas: Ethical, legal, social issues: for example, human subjects, HIPAA, informed consent, secondary use of data, confidentiality, privacy.”

*ii) Identify the Bloom’s verbs.*

The Bloom’s verbs are: “*Understanding”* and “*understand & apply”*.

*iii)* *Identify the content*.

Among the types of knowledge bioinformaticians need to understand and apply, ethical responsibilities, social issues, privacy, etc. are crucial. Thus, it may be necessary to have separate KSAs encompassing these knowledge areas. However, as stated, these two competencies cover impossibly broad content areas that no bioinformatician can be expected to fully “know”, understand, or apply; moreover, they are also time-dependent – i.e., key issues and responsibilities of today may change over time. Therefore, the final KSA is not likely to focus on *understanding* or *applying* specific ethical/legal/social content, and hence the wording of the KSA will need to reflect the intention of, rather than what is stated in, these competencies.

*iv) results of ii)b and iii) are entered into the first draft (“V1”) of KSAs; review V1 KSAs for redundancy.*

V1 KSA includes *understand* and *apply*, but the material/matters in question are both understood and applied in more contextual, rather than practical, ways by the practitioner – so would not necessarily be redundant with practical understand/apply KSAs that derive more directly from the scientific method KSAs. Therefore, a new KSA (about ethics, professionalism, privacy, etc.) is added to the list of V1 KSAs for consideration as the KSA-development process goes on.

***Phase 2****: Based on this version of KSAs, revise each so that it is observable, learnable, and assessable. Refine the KSAs so that they can be described with a systematic set of PLDs following Messick’s criteria.*

*v) Considering the Messick criteria, ensure that each KSA is distinct from others, is consistent with the overall intentions of the competency framers and plausible performance achievement.*

V1 KSAs should include subject matter specific to bioinformatics background understanding and application, and should also include a contextual dimension. This may be embedded into the PLDs. PLDs need to describe how ethical practice is observed, and how what is observed changes over the practitioner’s evolution.

*vi) Subject-matter experts confirm that KSAs describe what is required for the domain, and* *do represent observable, learnable, and assessable behaviors that are relevant.*

V2 KSAs include learnable/improvable and assessable attributes of ethical practice within other KSAs: transparency, rigour, and reproducibility are all essential features of ethical practice and these are natural parts of the scientific method. A separate KSA for ethical practice may hence be redundant.

*vii) PLDs are initially drafted for each KSA.*

The PLDs for V2-V3 KSAs focus on domain-specific knowledge: computational sciences, biological sciences, and the methods that are essential to bioinformatics practice. The more sophisticated practitioner is described as prioritizing ethical performance of each KSA. Once the PLDs for the semi-final list of KSAs are completed, however, subject-matter experts recognize that KSA performance that prioritizes transparency, rigour, and reproducibility - no matter how pervasive - does not capture the original *contextual* considerations of ethical practice (such as considering professionalism, avoiding bullying, protecting data confidentiality, etc.) that were present in the original competencies. Therefore, the “*Ethical practice*” KSA considered in V2 is reinstated. PLDs are drafted, describing a distinct developmental path *within* this KSA and levels of performance that are consistent *across* all KSAs. These PLDs are reviewed to ensure no overlap with other KSA PLDs; none is found. The final KSA list will therefore include one for ethical practice, and all PLDs for the other KSAs can retain their emphases on rigour, reproducibility, and transparency – covering relevant topical areas without adding redundancy.

Table B. Bioinformatics and biomedical informatics competencies.

| **COMPETENCIES** |
| --- |
| **Bioinformatics (Welch et al., 2014** [3]**)** |
| 1. An ability to apply knowledge of computing, biology, statistics and mathematics appropriate to the discipline. |
| 2. Knowledge of general biology, in-depth knowledge of at least one area of biology, and understanding of biological data-generation technologies. |
| 3. An ability to analyze a problem, and identify and define the computing requirements appropriate to its solution. |
| 4. An ability to apply mathematical foundations, algorithmic principles and computer science theory to the modeling and design of computer-based systems in a way that demonstrates comprehension of the trade-offs involved in design choices. |
| 5. An ability to design, implement and evaluate a computer-based system, process, component or program to meet desired needs in scientific environments. |
| 6. An ability to apply design and development principles in the construction of software systems of varying complexity. |
| **7.** An ability to use current techniques, skills and tools necessary for computational biology practice. |
| 8. An ability to function effectively on teams to accomplish a common goal. |
| 9. An understanding of professional, ethical, legal, security and social issues and responsibilities. |
| 10. An ability to communicate effectively with a range of audiences. |
| 11. An ability to analyze the local and global impact of bioinformatics and genomics on individuals, organizations, and society. |
| 12. Recognition of the need for and an ability to engage in continuing professional development. |
| 13. Detailed understanding of the scientific discovery process and of the role of bioinformatics in it. |
| 14. An ability to apply statistical research methods in the contexts of molecular biology, genomics, medical, and population genetics research. |
|  |
| ***Doctoral* biomedical informatics (Kulikowski et al., 2015** [2]**; emphasis added)** |
| **Fundamental scientific skills** |
| 15. Acquire professional perspective: understand and analyze the history and values of the discipline, and its relationship to other fields, while demonstrating an ability to read, interpret and critique the core literature. |
| 16. Analyze problems: analyze, understand, abstract and model a specific biomedical problem in terms of data, information and knowledge components. |
| 17. Produce solutions: use the problem analysis to identify and understand the space of possible solutions, and generate designs that capture essential aspects of solutions and their components. |
| 18. Articulate the rationale: defend the specific solution and its advantage over competing options. |
| 19. Implement, evaluate and refine: carry out the solution (including obtaining necessary resources and managing projects), evaluate it, and iteratively improve it. |
| 20. Innovate: create new theories, typologies, frameworks, representations, methods and processes to address biomedical informatics problems. |
| 21. Work collaboratively: team effectively with partners within and across disciplines. |
| 22. Educate, disseminate and discuss: communicate effectively to students and to other audiences in multiple disciplines in persuasive written and oral form. |
| **Scope and breadth of the discipline** |
| 23. Prerequisite knowledge and skills: students must be familiar with biological, biomedical and population-health concepts and problems, including common research problems. |
| 24. Fundamental knowledge: understand the fundamentals of the field in the context of the effective use of biomedical data, information and knowledge: e.g., biology (molecule, sequence, protein, structure, function, cell,  tissue, organ, organism, phenotype, populations); translational and clinical research (genotype, phenotype, pathways, mechanisms, sample, protocol, study, subject, evidence, evaluation); healthcare (screening, diagnosis (diagnoses, test results), prognosis, treatment (medications, procedures), prevention, billing, healthcare teams, quality assurance, safety, error reduction, comparative effectiveness, medical records, personalized medicine, health economics, information security and privacy); personal health (patient, consumer, provider, families, health promotion, personal health records); population health (detection, prevention, screening, education, stratification, spatio-temporal patterns, ecologies of health, wellness). |
| **Procedural knowledge and skills** |
| 25. For substantive problems related to scientific inquiry, problem solving and decision making, apply, analyze, evaluate and create solutions based on biomedical informatics approaches. |
| 26. Understand and analyze complex biomedical informatics problems in terms of data, information and knowledge. |
| 27. Apply, analyze, evaluate and create biomedical informatics methods that solve substantive problems within and across biomedical domains. |
| 28. Relate such knowledge and methods to other problems within and across levels of the biomedical spectrum. |
| **Theory and methodology** |
| 29. Theories: understand and apply syntactic, semantic, cognitive, social and pragmatic theories as they are used in biomedical informatics. |
| 30. Typology: understand and analyze the types and nature of biomedical data, information and knowledge. |
| 31. Frameworks: understand and apply the common conceptual frameworks used in biomedical informatics: including, e.g., belief networks, programming approach (e.g., object-oriented programming), representational scheme (e.g., problem-space models), or an architectural design (e.g., Web services). |
| 32. Knowledge representation: understand and apply representations and models that are applicable to biomedical data, information and knowledge: e.g., knowledge representation is a method of encoding concepts and relationships in a domain using definitions that are computable (e.g., first-order logics). |
| 33. Methods and processes: understand and apply existing methods (e.g., simulated annealing) and processes (e.g., goal-oriented reasoning) used in different contexts of biomedical informatics. |
| **Technological approach** |
| 34. Prerequisite knowledge and skills: assumes familiarity with data structures, algorithms, programming, mathematics, statistics. |
| 35. Fundamental knowledge: understand and apply technological approaches in the context of biomedical problems: e.g. imaging and signal analysis; information documentation, storage and retrieval; machine learning, including data mining;  networking, security, databases;  natural language processing, semantic technologies; representation of logical and probabilistic knowledge and  reasoning;  simulation and modeling;  software engineering. |
| 36. Procedural knowledge and skills: for substantive problems, understand and apply methods of inquiry and criteria for selecting and utilizing algorithms, techniques and methods. |
| 37. Describe what is known about the application of the fundamentals within biomedicine. |
| 38. Identify the relevant existing approaches for a specific biomedical problem. |
| 39. Apply, adapt and validate an existing approach to a specific biomedical problem. |
| **Human and social context** |
| - Design: e.g., human-centered design, usability, human  factors, cognitive and ergonomic sciences, and engineering. - Evaluation: e.g., study design, controlled trials, observational studies, hypothesis testing, ethnographic methods, field observational methods, qualitative methods, mixed methods. - Social, behavioral, communication and organizational sciences:  e.g., computer-supported cooperative work, social networks, change management, human-factor engineering, cognitive task analysis, project management. - Ethical, legal, social issues: e.g., human subjects, HIPAA, informed consent, secondary use of data, confidentiality, privacy. - Economic, social and organizational context of biomedical research, pharmaceutical and biotechnology industries, medical instrumentation, healthcare, and public health. |
| **Procedural knowledge and skills** |
| 40. Apply, analyze, evaluate and create systems approaches to the solution of substantive problems in biomedical informatics. |
| 41. Analyze complex biomedical informatics problems in terms of people, organizations and socio-technical systems. |
| 42. Understand the challenges and limitations of technological solutions. |
| 43. Design and implement systems approaches to biomedical informatics applications and interventions. |
| 44. Evaluate the impact of biomedical informatics applications and interventions in terms of people, organizations and socio-technical systems. |
| 45. Relate solutions to other problems within and across levels of the biomedical spectrum. |

**References**

1. Clark R, Feldon D, van Merriënboer J, Yates K, Early S. Cognitive Task Analysis. In: Spector JM, Merrill MD, Elen J, Bishop MJ, editors. Handbook of research on educational communications and technology. 3rd ed. Mahwah, NJ: Lawrence Earlbaum Associates; 2008. pp. 577–593.

2. Kulikowski CA, Shortliffe EH, Currie LM, Elkin PL, Hunter LE, Johnson TR, et al. AMIA Board white paper: Definition of biomedical informatics and specification of core competencies for graduate education in the discipline. J Am Med Informatics Assoc. 2012;19: 931–938. doi:10.1136/amiajnl-2012-001053

3. Welch L, Lewitter F, Schwartz R, Brooksbank C, Radivojac P, Gaeta B, et al. Bioinformatics Curriculum Guidelines: Toward a Definition of Core Competencies. PLoS Comput Biol. 2014;10: e1003496. doi:10.1371/journal.pcbi.1003496
