# Supplementary material for: The Mastery Rubric for Bioinformatics: A tool to support design and evaluation of career-spanning education and training
Source: PLoS One. 2019 Nov 26;14(11):e0225256. doi: 10.1371/journal.pone.0225256 (PMC6879125; doi:10.1371/journal.pone.0225256)
Supplement: Attachment — Submitted filename: Responses to Reviewers_R1_TA_26Sept.docx [file pone.0225256.s002.docx]

Responses to Reviewers

PONE-D-19-15839
The Mastery Rubric for Bioinformatics: supporting design and evaluation of career-spanning education and training
PLOS ONE

NB: we have added “TOOL” to the title

New title: The Mastery Rubric for Bioinformatics: a tool to support design and evaluation of career-spanning education and training

**Reviewer #1**: The authors describe the process of creating and validating a mastery rubric for bioinformatics training programs. The validation was thoroughly checked; they assessed whether it aligned with previously identified bioinformatics competencies as well as the principles of andragogy. The authors explain the problems that have been encountered while trying to create bioinformatics curricula using currently available bioinformatics competencies and discuss how this mastery rubric will solve some of these challenges. This paper is useful for anyone creating a training program in bionformatics. It's scope also extends beyond that, and is a concrete example of how mastery rubrics can be created and of general use for any program/discipline.

To increase the readability (which will affect the impact of this paper) there are two main concerns that should be addressed:

*1. The authors use far far too many acronyms. I understand acronyms reduce manuscript length, however, they are difficult for the reader to remember and thus negatively influence readability of the manuscript. The number of acronyms in this paper resulted in a much greater reading time for this manuscript compared to other manuscripts of similar length that I have read in the past. I also had to resort to drafting a acronym cheatsheet for myself so I could read this coherently.*

**We introduce 3 acronyms in the abstract, KSAs, PLDs and the MR-Bi. These are the only ones we kept and all other acronyms are now spelled out. We retained J1 and J2 for our two Journeyman levels but put “Journeyman” in wherever J1 or J2 appear. We left the FAIR acronym in Table 1 (because it would make that PLD particularly unwieldy and there is a footnote for resolving the acronym very close by (in the cell below).**

*2. In many instances in this manuscript, sentences are overly long and complex.*

**We have reviewed the document, streamlining wherever possible.**

**Reviewer #2**: In this manuscript entitled “The Mastery Rubric for Bioinformatics: supporting design and evaluation of career- spanning education and training”, Tractenberg and colleagues have submitted and summarized an important quantity of work dealing with the aspect of developing and describing a metric for the development of a bioinformatics education (undergraduate and graduate) and training (later specialized workshops) programs. The submission is well written, in a slightly dense format, with well written english.

The methodology used involved the development of a Mastery Rubric for Bioinformatics, created to address the challenges of integrating competencies into actionable teaching and learning goals. To do this they defined “Knowledge, Skills and Abilities” (KSAs) that are generic enough to be used in a number of scientific fields, but are specific here to the challenge of measuring and evaluating bioinformatics skills. These can then be used ro generate curricula for various levels of bioinformatics training.

Concerns:

*1. The way the data was gathered, used and analyzed is described in some details, but it would be very useful to also add a figure presenting the workflow of this process. Also maybe abstracting the figure from one of the annexes.*

**We appreciate the reviewer’s suggestion. The figures in the supplementary materials were, in fact, originally proposed for the main text; but the workflows represented in both the cognitive task analysis and the overall KSA and PLD drafting process seemed to require much more discussion with examples than a simple abstract could provide. We ultimately decided that the (narrative) summary of the results from these workflows would suffice for the main text, and then we elaborated the workflow with comprehensive descriptions for the supplementary materials. Our purpose was to keep the reader following the line of argument without getting bogged down in the details of psychometric standard setting. We have now more clearly labeled this as the workflow, and directed the interested reader to the figure/explanation and examples in the supplementary materials.**

*2. Although this was by their own words not a quantitative analysis, some numbers on how many shared KSAs that characterize the scientific method were used, that were later shown to be “essential to bioinformatics education and training”.*

**We added the list of KSAs (I had miscounted, there are 8) that represent the scientific method to the KSA results (now on p. 10 of the manuscript). In fact, the KSAs for bioinformatics and for “scientific thinking”, captured in a new version of the first (2010) Mastery Rubric, are identical except that “scientific thinking” includes only one KSA about prerequisite knowledge (bioinformatics includes two separate ones). The new version of the original Mastery Rubric is included in a forthcoming book about the Mastery Rubric construct itself, which is in the final writing stage, however not yet published and hence not yet citable).**

*3. This is presented as a “tool”, but it is clear no tool is presented. How an academic needs to work through this decision tree to see how her or his course is aligned to the KSAs and the various career stages is not clearly laid-out. A bit more of a a description how this could be best performed needs more details.*

**The Mastery Rubric for Bioinformatics itself, Table 1, is the tool that is presented in this manuscript. However, we do discuss the Mastery Rubric construct as a tool, as well. We added “tool” to clarify that the MR-Bi is the tool to the title, abstract, end of introduction, and discussion.**

**We debated how best to present the many ways that this particular Mastery Rubric could be utilized, but in the end decided that there were too many to put in the discussion of this paper – since it was intended to describe the tool. We noted, in the final sentence, that, “Specifics on how to accomplish these are the topics of our ongoing work”, and we are currently writing a book about how to use the MR-Bi. The MR-Bi has been discussed with bioinformaticians in New Zealand, the United States, and Europe, with the first formal workshop on how to create learning outcomes using the tool given in Stockholm September 2019. This work is truly ongoing, but elaborating on the methods that we allude to in the manuscript would both make the paper extremely long, and also would detract from our purposes in introducing the MR-Bi in this paper so that the bioinformatics community can become more familiar with it as we continue to develop robust documentation of the different methods for use.**

**We also added a point to the end of the introduction to clarify that this paper’s purpose is to present the MR-Bi, and that specific uses are the topic of ongoing work.**

*4. Adoption of this method in real life should have also been described, if it exists.*

**The paper is focused on the MR-Bi itself and does not include anecdotes about individuals who have used it (there are a few around the world who have worked with it). We have clarified our suggestions for possible applications, some of which have been explored in workshops to date. We have also reiterated our commitment to ongoing work to demonstrate how to use the MR-Bi, and we will continue to promote and encourage adoption of this method through our various networks (NBIS, GOBLET, ELIXIR, GEA, etc.) around the world.**

Editor:

*I agree with both reviewers in their assessment that the manuscript is somewhat dense and that a lot of acroynms are used. Perhaps it would make for an easier read if "KSA" were replaced with compentencies throughout and when KSA is mentioned on page 17 (with [22] cited) you could say something like "(KSAs, hereafter referred to as competencies)" or similarly for PLDs on Page 20 "(PLDs; [36], hereafter referred to as descriptors)". Likewise, in Table 2, I think you can spell out prerequisite knowledge instead of having PK in the table header. These are just a few examples for improvement. I would also recommend a figure as Reviewer 2 suggests, for helping readers to understand how your MR-Bi was arrived at.*

**As noted in response to reviewer 1,** **we introduce 3 acronyms in the abstract, KSAs, PLDs and the MR-Bi. These are the only ones we kept and all other acronyms are now spelled out. PK was spelled out in Table 2 (all acronyms were removed).**

**As noted in response to reviewer 2, we had originally planned figures for the workflows, but the could not be summarized succinctly and whenever we tried to integrate a figure outlining the formal psychometric standard setting without accompanying details (because it is a series of formal methods), the figure didn’t make sense. We opted to include the figure and its full description in the Supplemental materials, to ensure both that interested readers could find this additional information, and that uninterested readers would not be distracted by it.**
